# Resveratrol, lunularin and dihydroresveratrol do not act as caloric restriction mimetics when administered intraperitoneally in mice

**DOI:** 10.1038/s41598-019-41050-2

**Published:** 2019-03-14

**Authors:** Kathrin Pallauf, Dawn Chin, Ilka Günther, Marc Birringer, Kai Lüersen, Gerald Schultheiß, Sarah Vieten, Jürgen Krauß, Franz Bracher, Nicolas Danylec, Sebastian T. Soukup, Sabine E. Kulling, Gerald Rimbach

**Affiliations:** 1 Institute of Human Nutrition and Food Science, University of Kiel, Hermann-Rodewald-Straße 6, 24118 Kiel, Germany; 2 Department of Nutritional, Food and Consumer Sciences, Fulda University of Applied Sciences, Leipziger Straße 123, 36037 Fulda, Germany; 3 Animal welfare office, University of Kiel, Olshausenstraße 40, 24118 Kiel, Germany; 4 Department of Pharmacy, Center for Drug Research, Ludwig-Maximilians University, Butenandtstraße 5-13, 81377 Munich, Germany; 5 Max Rubner-Institut, Federal Research Institute of Nutrition and Food, Department of Safety and Quality of Fruit and Vegetables, Haid-und-Neu-Straße 9, 76131 Karlsruhe, Germany

## Abstract

Resveratrol as well as caloric restriction were shown to extend lifespan in some model organisms and may possibly delay onset of ageing-related diseases in humans. Yet, resveratrol supplementation does not always extend lifespan of animal models or improve health status of humans. Because of interindividual differences in human microbiota, resveratrol metabolite production in the gut differs. While some individuals produce lunularin and dihydroresveratrol in their gut, others produce dihydroresveratrol only. Therefore, we addressed the question whether these metabolites differ in their biological impact on ageing and intraperitoneally injected 13-month-old C57BL/6JRj mice on an *ad-libitum* (AL) HFD with resveratrol, dihydroresveratrol or lunularin (24 mg/kg bodyweight; 3 times/week). Compared to mice injected with vehicle (AL-control), resveratrol and dihydroresveratrol did not change bodyweight and had no impact on insulin or glucose levels while lunularin slightly reduced feed intake and bodyweight gain. CR-mice showed lowered cholesterol, insulin and leptin levels, elevated adiponectin and phosphorylated AMPK levels in liver as well as increased transcription of *Pck1 and Pgc1α* when compared to the AL-control. In contrast, injections with the test substances did not change these parameters. We therefore conclude that in our model, resveratrol, lunularin and dihydroresveratrol did not act as CR mimetics.

## Introduction

Since Howitz, *et al*.^1^ showed that *trans*-resveratrol (hereafter referred to as resveratrol) improved lifespan of yeast, this secondary plant metabolite has been studied extensively as potential anti-ageing drug in model organisms and clinical trials^2^. Its impact on lifespan has been compared to a calorically restricted diet (CR)^3^. CR appears to extend lifespan in some model organisms and to decrease blood pressure, insulin and cholesterol in humans^4^. Thus, CR could possibly delay the onset of ageing-related diseases.

In mice, compared to an *ad-libitum* diet, CR improved insulin and leptin sensitivity and lowered cholesterol levels while lifespan was lengthened^5,6^. Furthermore, in the liver of fasting mice, transcription of genes involved in mitochondrial biogenesis and glucose metabolism such as the peroxisome proliferator-activated receptor gamma coactivator 1-alpha (*Pgc-1α*), the NAD-dependent deacetylase sirtuin-1 (*Sirt1)*, the mitochondrial pyruvate dehydrogenase lipoamide kinase isozyme 4 (*Pdk4*) and the phosphoenolpyruvate carboxykinase (*Pck1*) was elevated^7,8^. Additionally, the adenosine monophosphate-activated kinase (AMPK) is induced by fasting since falling ATP levels lead to an elevated AMP/ATP ratio. The AMPK activates glycolysis, autophagy and inhibits fatty acid synthesis^9^. In contrast, major urinary proteins (MUPs), which trigger adaptive behaviour in mice via scent communication and affect glucose and lipid metabolisms^10^ are down-regulated when nutrient supply is decreased^11,12^.

Somewhat similar to CR, in mice fed resveratrol as part of a high fat diet, insulin sensitivity was improved, bodyweight and fat mass were lowered and the mice lived longer than the non-supplemented controls^13,14^. Moreover, in mice, resveratrol was shown to activate enzymes thought to benefit longevity such as the deacetylase SIRT1 and AMPK^15^ and to induce nuclear factor (erythroid-derived 2)-like 2 (NRF2) target gene heme oxygenase (*Hmox*1) which is involved in cellular stress response^16^. Another transcription factor that mediates stress response, forkhead box protein O3 (FOXO3), was also induced in resveratrol-supplemented mice^17^. Interestingly, human SNPs in FOXO3 that enhance its expression have been related to longevity^18^. FOXO3 also stimulates its own transcription and is inhibited by growth factors^19^. An additional mechanism by which resveratrol could promote healthy ageing is by attenuating ageing-related increase in inflammation. In old mice, resveratrol reduced transcription and levels of pro-inflammatory cytokines such as tumour necrosis factor alpha (*Tnfα)*^20^. Furthermore, dietary resveratrol may decrease obesity and therefore the incidence of related diseases is via inhibiting sodium-dependant glucose transporter 1 (SGLT1)-mediated glucose uptake in the gut^21^. Additionally, resveratrol was shown to influence gut microbiota by increasing the Bacteroidetes-to-Firmicutes ratios correlating negatively with bodyweight^22^.

Substances that mimic the positive effects of CR on health and longevity have been referred to as caloric restriction mimetics (CRMs) and resveratrol has been repeatedly discussed as being such a CRM^23^. In a review article written at the National Institute of Aging (U.S.), the concept of a CRM has been specified as a substance mimicking “the metabolic, hormonal, and physiological effects of CR” without “significantly reduc[ing] long-term food intake”. Additionally “it activates stress response pathways observed in CR and provides protection against a variety of stressors; and it produces CR-like effects on longevity, reduction of age-related disease and maintenance of function”^24^.

Many CRM candidate substances have shown serious side effects. For example, the inhibitor of the cell-growth- and proliferation-regulating kinase mTOR rapamycin is an immunosuppressant and 2-deoxyglucose, which inhibits glycolysis, was cardiotoxic in rats when administered chronically^25,26^. In contrast, chronic resveratrol intake seems to be safe as stated by the European Food and Safety Authority^27^. However, not all studies observed a positive impact of resveratrol on lifespan and therefore do not support a role of resveratrol as putative CRM. On the one hand, C57BL/6 mice on a high fat diet lived longer when supplemented with resveratrol^13^. On the other hand and similar to other polyphenols and polyphenol-containing plant extracts^4^, in genetically heterogeneous mice on a standard fat diet at a concentration of 0.03% or 0.12% feed, resveratrol did not show lifespan extension^28^. Thus, diet and genetic background seem to influence the effect of resveratrol on lifespan extension. Interestingly, there have been study outcomes pointing to the notion that lifespan prolongation by CR is more apparent in model organisms that are adapted to laboratory conditions (and possibly overfed) than in non-model organisms^29^. Taken together with the finding that resveratrol could only prolong lifespan when fed as part of a high fat diet^13,30^, this could imply that resveratrol counteracts the negative effects of elevated energy intake.

**Figure 1:**
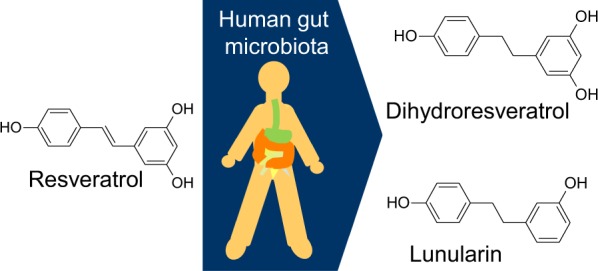
Chemical structures of resveratrol and its metabolites dihydroresveratrol and lunularin which are produced by human gut microbiota.

Clinical trials also show contradicting outcomes in healthy human subjects as well as patients with ageing- and/or overweight-related conditions^31,32^. Interestingly, Bode, *et al*.^33^ showed that interindividual differences in gut microbiota influenced resveratrol metabolism (Fig. 1). They identified three different metabolite profiles that differed in the presence of a reduced and dehydroxylated metabolite of resveratrol (lunularin). While all human trial participants were able to produce the reduced metabolite of resveratrol (dihydroresveratrol) in their gut, only some could produce lunularin. Furthermore, the lunularin-producing group could be divided into high and low producers.

Hence, we wanted to study the possibility that differing gut microbiota compositions influence the study outcome of trials with resveratrol by using an animal model that had previously shown to benefit from stilbene supplementation.

We fed 12-month-old C57BL/6 mice with an *ad-libitum* high fat, high sugar diet. This model was chosen to imitate diet-induced obesity in middle-aged humans. At around 45–65 years of age, the incidence of ageing-related chronic diseases such as diabetes and hypertension increases in humans from so-called Western world countries^34–36^. Obesity further increases the risk of developing these diseases^37^ that, putatively, may be alleviated by resveratrol supplementation^38^.

To test our hypothesis that resveratrol, and the gut metabolites dihydroresveratrol and lunularin, acted differently on molecular targets involved in ageing, we injected the mice and thereby aimed at minimising contact of the tested bioactives with gut microbiota. As a control, we injected mice with the vehicle PEG/saline (*ad-libitum* – AL-control). We compared these mice with each other and with a group of mice that was fed CR and also injected i.p. with PEG/saline (CR-control).

In 2016, the EFSA concluded that resveratrol supplements up to 150 mg/day were safe for human use^27^. Thus, we chose a dose of approximately 10 mg resveratrol (or equimolar bioacatives)/day *kg mouse. Injecting the mice three times a week, this meant 24 mg/kg bodyweight. While dose scaling according to bodyweight would equal around 700 mg resveratrol/day for a 70 kg human, clearance ratios for xenobiotics were shown to rise with decreasing bodyweights. Applying allometric principles based on animal to human comparisons of total clearance rates, doses for humans that are extrapolated from mouse studies would be 5–10 lower than expected from mere conversion according to bodyweight^39^. Therefore, the dose applied to our mice would be comparable to 75–150 mg resveratrol per day in humans.

## Results

### Resveratrol and its metabolites are found in mouse liver

Resveratrol and the microbial metabolites dihydroresveratrol and lunularin were quantified in mouse liver samples by using a UHPLC-MS/MS method that meets the validation criteria of the FDA. In addition, 2 quality control samples (500 pmol/g liver analyte level) were measured together with the study samples to ensure proper analysis. The accuracies of these quality control samples were 99% and 100% for resveratrol, 97% and 99% for dihydroresveratrol and 113% and 111% for lunularin.

In the livers of the AL-control and CR-control neither resveratrol, nor dihydroresveratrol or lunularin were detected. In the resveratrol group only 5 out of 9 liver samples exhibited measurable, but low resveratrol levels (6.9 ± 4.0 pmol/g liver (mean ± SEM)), whereas dihydroresveratrol was present at significantly higher levels (230.0 ± 84.6 pmol/g liver (mean ± SEM)) in all these samples except one. This interesting finding indicates that resveratrol was efficiently metabolized to dihydroresveratrol in resveratrol-injected mice.

In mice receiving dihydroresveratrol, neither resveratrol nor lunularin was detected in the liver samples, whereas dihydroresveratrol was detectable in all samples from the dihydroresveratrol group with a mean level of 112.2 ± 30.0 pmol/g liver (mean ± SEM). Lunularin was only detectable in the lunularin group (28.8 ± 7.1 pmol/g liver (mean ± SEM)) but not in the groups receiving resveratrol or dihydroresveratrol.

### Mean feed intake and bodyweight of resveratrol injected mice does not differ from AL-control mice

**Figure 2:**
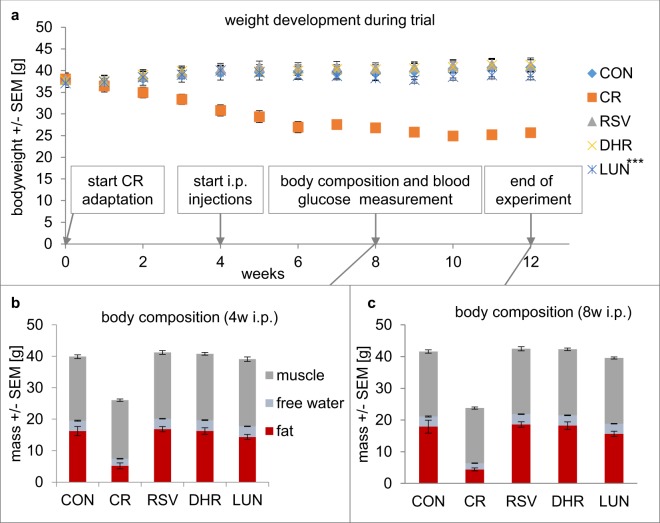
Weight development of C57BL/6 mice during the trial (**a**), body composition (halfway through the intraperitoneal injections (**b**) and at the end of the trial (**c**)). While we observed a typical CR phenotype when looking at bodyweight and body composition of CR mice, only the lunularin group showed a significant difference in bodyweight development compared to the AL control. Groups of mice: CON – AL-control, CR – CR-control, RSV – resveratrol, DHR – dihydroresveratrol, LUN – lunularin, w – weeks, n ≥ 8; ***p < 0.001 (ANCOVA, Dunnett AL-control).

Feed intake was checked daily and weight weekly. While i.p. injections with resveratrol or dihydroresveratrol did not lower feed intake (3.00 ± 0.05 g and 3.01 ± 0.10 g, respectively) injecting lunularin slightly lowered feed intake compared to PEG/saline-injected mice (2.81 ± 0.1 g, p = 0.048 Dunnet AL–control, Supplemental Data X1). This slightly lower feed intake could explain the lower than AL-control body weight of lunularin mice at the end of the trial. A regression analysis of weekly mouse bodyweights showed that the difference in weight development between lunularin and AL-control mice was highly significant (p < 0.001). Resveratrol-injected and dihydroresveratrol-injected mice did not differ from AL-control mice (p = 0.147 and 0.764, respectively, Fig. 2). We excluded the CR mice from the statistical analysis of feed uptake and bodyweight.

### CR but not resveratrol-injected mice have lower glucose and insulin levels in plasma than control mice on a high fat diet

**Figure 3:**
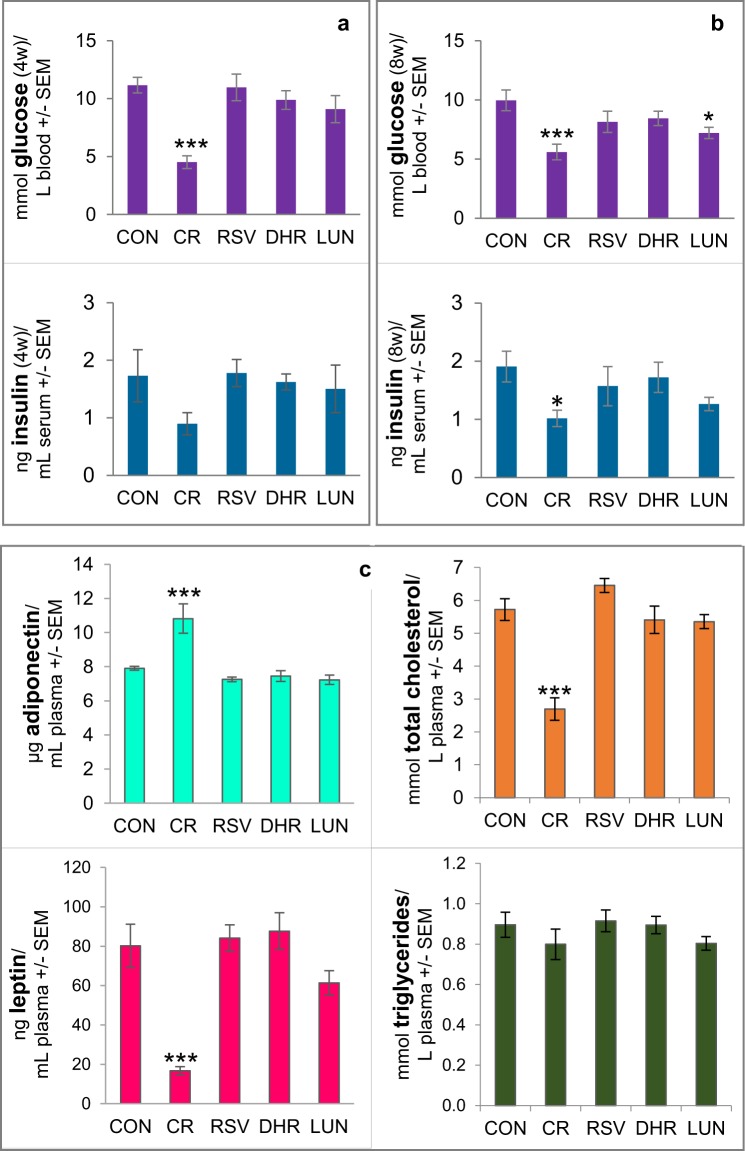
Insulin sensitivity was monitored by oral glucose tolerance tests halfway through (**a**) and at the end of the study. (**b**) It was improved by restricting feed intake (CR) but not by injecting resveratrol (RSV) nor dihydroresveratrol (DHR). Glucose and insulin levels in the blood collected from the facial vein of the mice are shown. (**c**) Leptin, adiponectin, total cholesterol and triglyceride concentrations in mouse plasma after 8 weeks of intraperitoneal injections with resveratrol, dihydroresveratrol, lunularin (LUN) or with vehicle (20% PEG) containing saline (CON). CR – injections with vehicle/saline and 40% CR. Leptin and cholesterol levels in CR mice were significantly lower and adiponectin was significantly higher in CR mice than in CON mice. Plasma levels of leptin, adiponectin, cholesterol or triglycerides in CON mice injected did not differ statistically from mice injected with resveratrol, dihydroresveratrol or lunularin. ***p < 0.001, *p < 0.05, linear model, Dunnett CON (AL-control), n ≥ 8, except insulin measurements (n = 5–10).

Insulin sensitivity was monitored by oral glucose tolerance tests at the beginning, halfway through and at the end of the study. Analyses of the blood collected 30 minutes after applying a glucose bolus via gavage (2 g/kg mouse) showed that injections with resveratrol had not changed glucose or insulin levels compared to the controls. However, mice on CR showed improved insulin sensitivity as measured by glucose and insulin levels. Interestingly, after 8 weeks of injections with lunularin, glucose levels were slightly lower than in the controls (p = 0.03, Fig. 3).

### CR but not resveratrol-injected mice had lower cholesterol and leptin plasma levels than control mice on a high fat diet

As expected, CR-feeding lowered leptin and total cholesterol levels compared to AL fed controls. In contrast, adiponectin was elevated by CR while triglyceride levels seemed unaffected (Fig. 3c). However, neither resveratrol nor its gut microbiota metabolites dihydroresveratrol or lunularin affected the hormones or lipids measured.

### Transcription of genes involved in Sirt1-signalling and glucose metabolism in mouse liver was affected by CR but not by i.p. administered resveratrol

**Figure 4:**
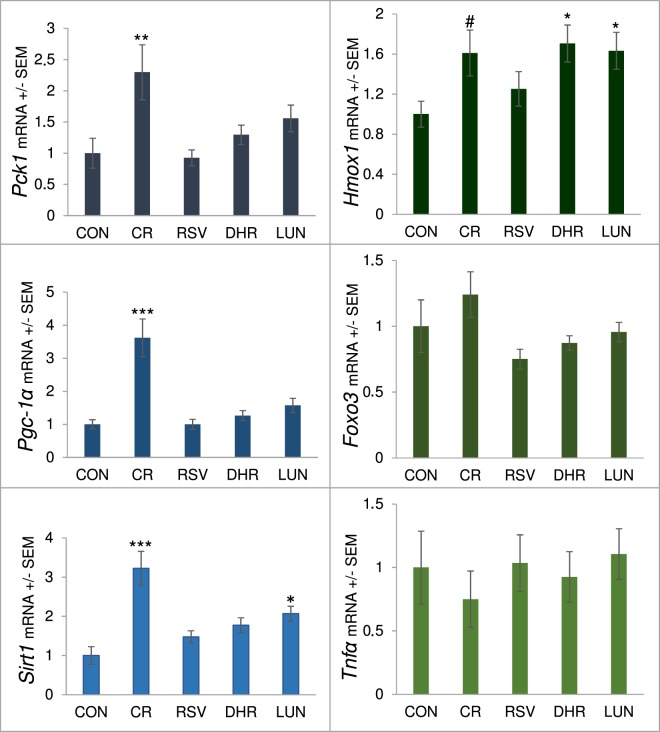
*Pck1*, *Pgc-1α*, *Sirt1*, *Hmox1*, *Foxo3* and *Tnfα* mRNA levels in liver from 15 month old C57BL/6JRj mice on an *ad-libitum* high fat diet after 8 weeks of intraperitoneal injections with resveratrol (RSV), dihydroresveratrol (DHR) or lunularin (LUN) compared to mice injected with vehicle (20% PEG) containing saline fed *ad-libitum* (CON) or 40% caloric restriction (CR). mRNA levels were normalised to *Gapdh* and *Actb*, related to CON and are shown +/− SEM. ***p < 0.001, **p < 0.01,*p < 0.05, ^#^p < 0.1, linear-mixed model, Dunnett CON (AL-control).

*Pgc1-α* mRNA, which is up-regulated under caloric restriction and may contribute to the life-prolonging effect of lowered calorie intake^3^, was elevated in our CR mice when compared to the controls. Since SIRT1 was shown to activate PGC-1α^7^ and resveratrol was shown to activate SIRT1 and PGC-1α^15^, we also measured *Sirt1* expression. While CR increased transcription, resveratrol did not significantly change *Sirt1* mRNA levels in the liver. Interestingly, lunularin also shows a smaller than CR but significant increase in *Sirt1* expression. Gluconeogenesis as measured by *Pck*1 expression appeared as not being influenced by resveratrol or resveratrol metabolite injections but by CR (Fig. 4).

### Transcription of longevity-associated Foxo3, cytokine Tnfα or Nrf2 target gene Hmox1 was not affected by i.p. administered resveratrol

NRF2 target gene *Hmox*1 was slightly induced by CR as well as by dihydroresveratrol and lunularin injections. However, resveratrol did not activate *Hmox*1 expression. mRNA levels of the transcription factor *Foxo3* did not appear to be affected by any of the bioactives injected. Similarly, *Tnfα* expression appeared unchanged within the groups of our trial (Fig. 4).

### While AMPK is induced by CR, resveratrol and its metabolites do not lead to elevated levels of phosphorylated AMPK in mouse liver

**Figure 5:**
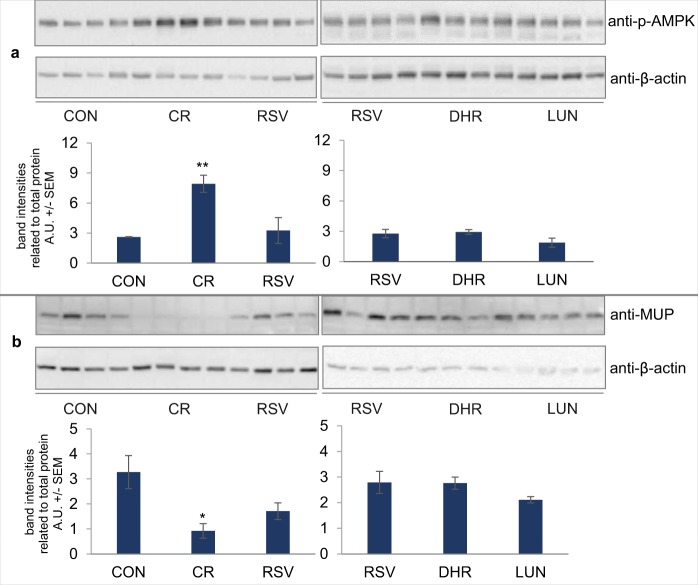
Mice on CR showed increased levels of pAMPK (**a**) while resveratrol (RSV)-, dihydroresveratrol (DHR)- and lunularin-injected mice (LUN) showed levels comparable to the AL-control mice (CON). In contrast, MUP levels (**b**) were lowered by CR but not by the compounds injected. Cropped western blot images. See Supplemental Figures for full-length blots. At least 4 representative mouse samples are shown. A.U. – arbitrary units, **p < 0.01,*p < 0.05, linear model, Dunnett CON (AL-control).

*In-vitro* studies have shown that resveratrol can activate AMPK and lead to its phosphorylation^13^ and, in accordance with Miller, *et al*.^5^, our mice on a CR diet showed elevated phospho-AMPK levels in liver. In contrast, in our mice injected with resveratrol, dihydroresveratrol or lunularin, we could not observe a rise of phosphorylated AMPK levels (Fig. 5a).

### Major urinary protein levels are lowered by CR but not by resveratrol

In the livers of mice injected with resveratrol, MUP levels were not significantly lowered compared to the control (Fig. 5b, left blot) and dihydroresveratrol- and lunularin-injected mice showed very similar MUP bands compared to resveratrol-injected mice (Fig. 5b, right blot). In contrast, in CR mice, MUP levels were approximately 30% of a*d-libitum* fed mice. The MUP antibody used did not distinguish between different MUP isoforms.

### Injections with resveratrol do not affect glucose transport in mouse ileum

**Figure 6:**
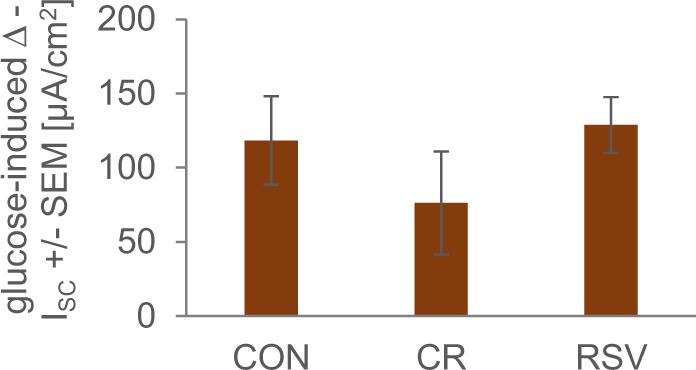
Intestinal glucose transport in the ileum from AL-fed mice and CR-fed mice injected with PEG/saline (CON and CR, respectively) compared to mice injected with resveratrol (RSV) as measured using Ussing chambers. There is no significant difference in the glucose-induced I_SC_ between resveratrol-injected mice and AL-control mice.

To examine whether CR or resveratrol treatment affects the intestinal active glucose transport, the ileum of the animals was mounted in Ussing chambers. As shown in Fig. 6, the glucose-induced I_SC_ determined for the ilea of the CR and resveratrol groups were not significantly different when compared to that of the controls.

## Materials and Methods

### Mice, diet and intraperitoneal injections

Eleven-month-old C57BL/6Rj mice were purchased from Janvier Labs, Saint-Berthevin, France and kept on a high fat, high sugar purified diet (18.7% protein, 21.1% fat, 13.4% starch, 32.9% sugar, 2.1 mg/kg cholesterol, Ssniff, Soest, Germany). The mice were housed individually in macrolon cages (type 3) in 55% relative humidity, 21–25 °C and with a 12 h light/dark cycle at the Food Science Institute at the University of Kiel. Mice were weighed weekly and fed daily. After a 6-week *ad-libitum* adaptation phase, the experiment began by dividing the mice into 5 groups with 10 mice each. Then, the calorically restricted group was fed 5% less than what the control group fed every 3 days until reaching −40% restriction. When CR had reached 40%, injections started. Resveratrol, dihydroresveratrol and lunularin were dissolved in PEG400 (Sigma-Aldrich Taufkirchen, Germany), at concentrations equimolar to 30 mg resveratrol/mL. This stock was diluted with saline (Braun, Melsungen, Germany) until reaching a PEG concentration of 20% v/v and injected at 24 mg resveratrol or equimolar amounts of dihydroresveratrol or lunularin/kg bodyweight. The control and CR mice were injected with 20% PEG400 in saline. Because of animal welfare laws, if individual CR mice lost more than 20% of their weight at the beginning of the experiment, they were fed up to 70% of what the AL controls consumed.

At the beginning, halfway through the experiment and prior to sampling the body composition of the mice was measured in a Bruker Minispec LF (Billerica, United States). Per experimental group, 8–10 mice were sacrificed at the end of the experiment. We lost 2 mice each in the control and the CR group and 1 mouse in the resveratrol group. Experimental procedures were carried out in accordance with German laws on animal protection (TierSchG §7–9). The experiment (V 242-27717/2016 (46-4/16)) was approved by the MELUR (state government of Schleswig-Holstein, Germany).

### Synthesis of resveratrol metabolites

Resveratrol for dihydroresveratrol synthesis and i.p. injections was purchased from Carl Roth (Karlsruhe, Germany). Dihydroresveratrol for i.p. injections was synthesized according to Faragher, *et al*.^40^. Lunularin (chemical purity >97%, determined by NMR) for i.p. injections and the analysis of resveratrol and its metabolites in mouse liver samples at the MRI Karlsruhe (Germany) was prepared as described previously in a modular synthesis^41^.

### Analysis of resveratrol and metabolites in mouse liver samples

The UHPLC-MS/MS analysis and the sample preparation for these analyses took place at the MRI, Karlsruhe, Germany. For a more exhaustive description, see Supplemental Methods. Mouse livers were homogenized with a ball mill (MM400; Retsch, Haan, Germany) spiked with internal standards and processed for UHPLC-MS/MS analyses on an QTrap 5500 mass spectrometer (AB Sciex, Darmstadt, Germany).The spectrometer was equipped with a Nexera LC system (Shimadzu, Duisburg, Germany) which consisted of a controller (CBM-20A), a degasser (DGU-20A5), two pumps (LC-30AD), an autosampler (SIL-30AC), a column oven (CTO-20AC) and a DAD (SPD-M20A). Separation of the analytes was achieved on a Waters Acquity HSS T3 (2.1 mm × 100 mm, 1.8 µm particle size) equipped with a pre-column (Waters Acquity HSS T3, 2.1 mm × 5 mm, 1.8 µm particle size) and a pre-in-line filter (Phenomenex Krudkatcher, 0.5 µm). The mobile phase consisted of an aqueous 25 mM ammonium formate buffer with 0.1% (v/v) formic acid and acetonitrile. The turbo spray ESI source of the MS was operated in the negative mode.

Analysis of UHPLC-MS/MS data was performed with the MultiQuant 2.1.1 software (ABSciex, Darmstadt, Germany). To obtain the calibration curves, the ratios of the analyte peak areas to the internal standard peak area were calculated and plotted against the analyte level. A best fit line was obtained by linear regression using a weighting of 1/x^2^. The method was validated based on FDA criteria.

For calculation of means and SEMs, values below the limit of detection (LOD) were set as 0.01 pmol/g liver and values between limit of quantitation (LOQ) and LOD were set as LOQ/2. n.d., not detected, values <LOD: resveratrol <2.0 pmol/g liver, dihydroresveratrol <2.3 pmol/g liver, lunularin <9.7 pmol/g liver.

### Body composition measurements

At the beginning, halfway through the experiment and prior to sampling, body composition was measured using the time-domain nuclear magnetic resonance technique (MiniSpec, Bruker, BioSpin MRI GmbH, Ettlingen, Germany) with the following parameters: X-ray energy settings: 45 kVp and 177 µA; voxel size: 76 µm; integration time: 300 ms; and projection setting: 250 projections per 180°. Fat mass, lean mass and free water weight were obtained within two minutes in the live animals.

### Blood parameters

Insulin levels were measured in serum using the Ultra Sensitive Mouse Insulin kit from Crystal Chem (Zaandam, Netherlands), leptin and adiponectin levels were measured in plasma using the mouse ELISA kits from R&D Systems (Abingdon, UK), total cholesterol was measured using the fluorimetric assay kit from Cayman Chemicals via Biomol (Hamburg, Germany) and triglycerides were measured using the Fluitest TG kit from Analyticon (Lichtenfels, Germany) all according the manufacturer’s instructions. Glucose levels in whole blood were measured using the Free Style glucometer from Abbott (Abbott Park, Illinois, United States).

### Oral glucose tolerance test

At the beginning, halfway through the experiment and prior to sampling, mice were fasted for 5 hours before receiving 2 g glucose/kg body weight via gavage. Thirty minutes later, blood was collected by puncturing the facial vein and glucose and insulin levels were determined. Glucose was purchased from Carl Roth (Karlsruhe, Germany) and dissolved at 0.5 g/mL in autoclaved tap water (Kiel, Germany).

### mRNA polymerase chain reaction

Liver total RNA was isolated using peqGold Trifast®(Peqlab, Erlangen, Germany). RNA concentration and purity were determined by measuring the absorbance at 230, 260 and 280 nm with a NanoDrop 2000^TM^ (Thermo Fisher, Waltham, USA). RNA aliquots were stored at −80 °C until PCR analysis. One-step quantitative reverse transcriptase PCR was carried out with the SensiMix™ SYBR No-ROX one step kit (Bioline, Luckenwalde, Germany) and with SybrGreen detection using the Rotorgene 6000 cycler (Corbett Life Science, Sydney, Australia). Relative mRNA levels of target genes were normalised to *Gapdh* and *Actb* gene expression and related to the AL-control. Mean value of expression in the AL-control group was set to an arbitrary unit of 1. All data are represented as means +/− SEM (n ≥ 8). See primers in Table 1.

**Table 1:** Primers used for quantitative reverse transcriptase PCR.

Primer	Forward (5′-…-3′)	Reverse (5′-…-3′)
*Actb*	GACAGGATGCAGAAGGAGATTACT	TGATCCACATCTGCTGGAAGGT
*Foxo3*	TTGTCCCAGATCTACGAGTGG	CCGTGCCTTCATTCTGAAC
*Gapdh*	CCGCATCTTCTTGTGCAGT	GGCAACAATCTCCACTTTGC
*Hmox1*	GAGCCTGAATCGAGCAGAAC	AGCCTTCTCTGGACACCTGA
*Pck1*	AGCCTTTGGTCAACAACTGG	TGCCTTCGGGGTTAGTTATG
*Pgc-1α*	TGCCCAGATCTTCCTGAACT	TCTGTGAGAACCGCTAGCAA
*Sirt1*	GTCTCCTGTGGGATTCCTGA	ACACAGAGACGGCTGGAACT
*Tnfα*	TCGTAGCAAACCACCAAGTG	AGATAGCAAATCGGCTGACG

### Western blotting

Cytosolic liver extracts were prepared by homogenizing fresh tissue in 10 mM HEPES (pH 7.9), 10 mM KCl, 1.5 mM MgCl_2_, 0.5 mM DTT, 0.1% Nonidet-P40, protease inhibitor cocktail and PhosSTOP^TM^ (all Sigma-Aldrich), leaving it on ice for 30 min with occasional vortexing before centrifuging at 4000×g and 4 °C for 5 min. Supernatant protein concentrations were determined with the BCA assay (Thermo Fisher Scientific, Schwerte, Germany). The samples were mixed with loading buffer, denatured at 95 °C for 5 min and separated on TGX Stain-Free Precast gradient gels (Biorad, Munich, Germany) and blotted onto a PVDF membrane. The membrane was blocked with 5% skim milk dissolved in TBS with 0.05% Tween-20 and probed with a primary antibody overnight (MUP antibody sc-21856, Santa Cruz Biotechnology Inc., Heidelberg, Germany; p-AMPK antibody CS-2523, Cell Signalling, Leiden, The Netherlands; Actin antibody ab3280, Abcam, Cambridge, UK) followed by a secondary antibody (anti-goat sc-2354: Santa Cruz Biotechnology Inc., Heidelberg, Germany, anti-mouse 170–5047; anti-rabbit 170–5046: Biorad). The bands were visualized with ECL reagent (Thermo Fisher Scientific) in a ChemiDoc XRS system (BioRad).

### Ussing chamber measurements

A segment of approximately 2 cm of the distal ileum (2–3 cm proximal to the ileocecal valve) was extracted from freshly sacrificed mice and transferred to ice-cold oxygenated KBR-buffer containing 115 mmol/L NaCl, 2.4 mmol/L K_2_HPO_4_, 0.4 mmol/L KH_2_PO_4_, 1.2 mmol/L MgCl_2_, 1.2 mmol/L CaCl_2_, and 25 mmol/L NaHCO_3_ (pH 7.4). Flushing and washing with ice cold KBR solution containing 5 µM indomethacin the segment was cut longitudinally along the remnants of the mesenteric attachment. Subsequently, the tissue was mounted in an Ussing chamber with a 0.3 cm^2^ exposed surface area (EasyMount chamber system with P2300 chambers and P2304 sliders; Physiologic Instruments, San Diego, USA). The Ussing chambers were filled with 5 mL KBR solution containing 10 mmol/L mannitol apically and 10 mmol/L glucose basolaterally, maintained at 37 °C and continuously bubbled with a hydrated mixture of 5% CO_2_/95% O_2_ (v/v). 0.1 µM tetrodotoxin was included in the serosal bath to minimize intrinsic residual neural activity. The transepithelial potential difference was continuously monitored under open circuit conditions using a DVC 1000 amplifier (WPI) and recorded through Ag-AgCl electrodes and KBR agarose bridges. Subsequently, the short-circuit current (ISC; μA cm^−2^) was measured via an automatic voltage clamp (VCC MC8 MultiChannel Voltage-Current Clamp; Physiologic Instruments, San Diego, CA, USA). Recordings were collected and stored using the A&A II (Acquire & Analyze Data) acquisition software (Physiological Instruments). Transepithelial resistance and ISC were allowed to stabilize for approximately 20 min. After that, 10 mM glucose were added apically to stimulate Na^+^-coupled glucose transport; 10 mM mannitol were given basolaterally. The glucose-stimulated ISC reached a stable plateau within 10 min. The ΔISC value that indicates intestinal SGLT-1 dependent glucose transport was calculated by the difference: ISC (with apical glucose) - ISC (without apical glucose).

### Statistics

The statistical software R^42^ was used to evaluate the data. For a more exhaustive description, see Supplemental Methods. Appropiate models were defined^43,44^. Based on graphical residual analyses the data was assumed to be approximately normally distributed. While for the blood parameters, feed intake and normalized mRNA levels an analysis of variances (ANOVA) was conducted, an analysis of covariances (ANCOVA) was conducted for the weight development^45,46^. These analyses were followed by multiple contrast tests (Dunnett)^47^.

## Discussion

Our study design included daily control of feed uptake and a controlled application of resveratrol and its metabolites. Moreover, we had AL fed mice as a negative control group and CR fed mice as a positive control group. Although we applied the substances intraperitoneally, we found the gut microbiota metabolite of resveratrol, dihydroresveratrol, in our resveratrol group.

While it is possible that we would have had more statistically significant results using a higher number of animals, we observed that many parameters described as being affected by resveratrol hardly changed or even (not significantly) tended to be further away from CR-control values than from AL-control values (for example, average bodyweight gain in resveratrol-injected mice was 3.3 g and 3.0 g in the AL-controls (Supplemental Data X-1)). Therefore, we do not think our non-significant results are a problem of statistical power. However, we did not carry out lifespan studies and only used one dose of resveratrol and its metabolites. Furthermore, our inbred strain used (C57BL/6Rj) might be less sensitive to resveratrol supplementation than more genetically heterogenous mice^48^ or other mouse genotypes^14^.

Interestingly, there is evidence in rodents and lower model organisms that resveratrol may not always extend lifespan or benefit health^28,49^. Negative results have also been reported for human trials supplementing resveratrol^31,50^.

Our CR mice showed a clear CR phenotype (lowered body weight, lowered glucose, insulin, leptin, cholesterol and elevated adiponectin)^11,51^ and we found changes in molecular targets typically affected by restricting feed intake such as MUP, pAMPK protein and *Pgc-*1α mRNA levels^5,11^. In contrast, injections with resveratrol or dihydroresveratrol did not affect bodyweight or the blood parameters measured compared to the control. Consistently, we did not observe differences in protein or mRNA levels measured in resveratrol- or dihydroresveratrol-injected mice.

Interestingly, injections with lunularin led to slightly lowered bodyweight and glucose levels in the mice. However, the mice fed less when injected with lunularin and this may be an indirect CR effect induced by lunularin lowering feed intake. This could also explain changes in blood parameters and mRNA expression that show the same tendency as CR and are significant for *Sirt1* in lunularin mice (Fig. 5).

While we found resveratrol and lunularin only in the mice that had been injected with the corresponding substance, we found dihydroresveratrol in resveratrol-injected mice. Furthermore, we found rather high inter individual variability in resveratrol and –metabolite levels. Resveratrol, dihydroresveratrol and lunularin liver levels at sampling depend on how fast the polyphenols were taken up after injection and on the rate of excretion. The rate of excretion depends largely on phase II metabolism that is known for large inter-individual variability^52^. Phenolic levels after oral administration in inbred mice showed standard deviations that were larger than the means^53^. In our mice, we find the highest variation in the dihydroresveratrol levels of resveratrol-injected mice. Here, we would expect to have even higher variability since these levels do not only depend on uptake and excretion but also on metabolization of resveratrol to dihydroresveratrol.

As far as we are concerned, murine metabolic enzymes that can reduce double bonds have not been identified. Therefore, finding dihydroresveratrol in resveratrol-injected mice points to the notion that resveratrol could be excreted into the gut, where its double bond is reduced by microbiota and, as a consequence, dihydroresveratrol is (re)absorbed. In line with this hypothesis, enterohepatic recirculation of resveratrol has been observed in rats before^54^. Alternatively, a liver reductase with affinity to resveratrol could have also produced dihydroresveratrol. However, further studies to clarify the origin of the dihydroresveratrol in resveratrol-injected mice were beyond the scope of this study.

The finding that resveratrol injections had no impact on insulin and glucose levels (and other parameters) stands in contrast to earlier findings^13,48^. Similar to Baur, *et al*.^13^, we used male ca. 1-year-old C57BL/6 mice on high fat diet. Conversely, we injected rather than fed resveratrol as part of the diet. Because of the different administration routes, resveratrol uptake doses are difficult to compare with each other. However, with 0.4 g resveratrol/kg feed and 72 mg resveratrol/(kg bodyweight week), assuming a feed intake per mouse per day of 3.5 g feed and a 35 g mouse, this would equal a daily intake per mouse of 1.4 mg via diet versus 0.36 mg i.p. Of interest, related to bodyweight, 1.4 mg resveratrol/35 g mouse would be 2.8 g of resveratrol for a human subject of 70 kg. However, interspecies extrapolation for micronutrient requirements or toxicological risk assessment is often flawed when merely using bodyweights. Applying allometric principles and taking different caloric demands of mice and humans into account, it is possible that, related to bodyweight, humans need an approximately 5–10-fold lower dose of resveratrol than mice for an effect^39,55^. Intake of resveratrol without further supplementation has been estimated to average 80–900 µg/day^56,57^. Yet, administering higher doses of resveratrol and its metabolites to our mice might have had an impact on insulin signalling. Furthermore, our diet had around 40% calories from fat while Baur and colleagues fed 60% calories from fat. In addition, our trial was shorter than the lifespan trial by Baur, *et al*.^13^. While resveratrol has been shown to affect ageing-related parameters after short term feed supplementation, in mouse strains other than C57BL/6, glucose tolerance was only slightly affected by resveratrol feed supplementation for 6 or 15 weeks^14^ or not at all^48^. Although we assume that at least part of the resveratrol we applied i.p. located to the gut because of enterohepatic circulation^54^, injecting rather than feeding resveratrol might have diminished effects of resveratrol on the microbiota or the intestinal sodium-dependent-glucose transport. When fed, resveratrol concentration in the gut is quite high and exposure is likely increased because it may re-enter the gut after uptake^54^. Here, it may interfere with glucose and alanine uptake via the SGLT1^58^ without affecting intestinal SGLT1 expression^59^. However, glucose uptake by SGLT1 was shown to be largely influenced by postranslational modification of the transporter and less by mRNA levels^60,61^. We did not observe differences in glucose uptake between intestines from resveratrol-injected mice and vehicle-injected mice (Fig. 6). Moreover, intestinal glucose transport of CR and CON mice did not differ significantly, possibly due to rather high inter individual deviations. However, higher glucose transport in the intestines of mice on long-term CR has been reported before^62^. Thus, it is possible that some type of adaptation leads to increased glucose transport and thereby, in part, compensates for low glucose feed intake that occurs in mice with limited nutrient supply.

In our settings, resveratrol did not alter transcription of genes previously described as responding to CR or the stilbene itself. This included transcription factors and their target genes as well as genes involved in metabolism (*Pck1*, *Pgc-1α*, *Sirt1*, *Hmox1*, *Foxo3* and *Tnfα*).

A reason for our contradicting data could be the timepoint we terminated the experiment. In a study by Fusser, *et al*.^16^, *Hmox1* expression was only elevated after short term application (7 days) of resveratrol and not after 6 months. However, the microbial metabolites lunularin and dihydroresvertrol activated *Hmox1* in our settings but resveratrol did not (p = 0.8). This is interesting because we find dihydroresveratrol also in the liver of resveratrol-injected mice.

*Tnfα* downregulation in mice liver was observed in 24 month old mice after 6 months of supplementation^20^ while our mice were almost 10 months younger. Although resveratrol was shown to activate the SIRT1 homologue SIR2 in yeast^1^, the stilbene may not act via PGC-1α^14^ or even activate mitochondrial biogenesis in rodents^63^.

Our finding that injections with resveratrol did not affect AMPK phosphorylation in mouse liver (Fig. 5a) is somewhat interesting since resveratrol has been described as activating AMPK and its action as being dependent on AMPK^6^. However, in the study cited, resveratrol was supplemented to the diet and the mice were younger than ours. Furthermore, Um and colleagues focussed on skeletal muscle and, also in contrast to our findings (Fig. 2b,c), found lowered fat mass in resveratrol-treated mice.

In liver homogenates from mice fed CR, MUP levels were considerably lower than in *ad-libitum* fed mice. There are various, highly polymorphic MUP isoforms. MUPs bind to pheromones and are excreted into urine where the pheromones are released from the MUPs in urine marks^64^. MUPs are central for interindividual recognition and reproduction in mice^65^. MUP expression is affected by diet and MUP1 may counteract insulin resistance and inhibit expression of gluconeogenic genes^66^. On one hand, mice on CR showed lowered MUP1 and MUP5 levels^6,12^, on the other hand, MUP levels were also reduced by high fat diet^10^ For mice on a high fat diet supplemented with resveratrol, up-regulation of MUP 1 and MUP 3 mRNA levels in liver has been reported^13^. While we could not distinguish between different MUP isoforms measuring protein levels via Western blot, we found reduced MUP in mice that fed 60% of a high fat diet compared to the AL-control. Injecting resveratrol (or resveratrol metabolites) did not affect MUP levels compared to animals injected with vehicle and saline (Fig. 5b).

Taken together, injecting C57BL/6 mice on a high fat diet with resveratrol or dihydroresveratrol, in contrast to CR-feeding, did not affect body weight, glucose, insulin, adiponectin, leptin or cholesterol blood levels, did not lower hepatic *Pck1*, *Pgc1α* or *Sirt1* mRNA levels and did not change phosphorylated AMPK or MUP protein levels in liver. For reasons, of which identifying the underlying mechanisms was beyond the scope of our study, lunularin lowered feed intake and, possibly as a consequence, body weight. Decreased feed intake (and therefore not lunularin itself) is likely to have caused lowered glucose and *Sirt1* mRNA levels. However, we cannot rule out that lunularin activated molecular targets leading to these outcomes.

By comparing our negative data for resveratrol as CRM with literature data from model organisms and human trials, we conclude that resveratrol may only exert its putative health effects under certain circumstances. Therefore, caution should be warranted when promoting resveratrol as caloric restriction mimetic.

## Supplementary information

Supplementary data, methods and figures

## Data Availability

All data generated or analyzed during this study is included in this published article (and its Supplementary Information files).

## References

[1] Howitz KT (2003). Small molecule activators of sirtuins extend Saccharomyces cerevisiae lifespan. Nature.

[2] Pallauf K, Rimbach G, Rupp PM, Chin D, Wolf IM (2016). Resveratrol and lifespan in model organisms. Current medicinal chemistry.

[3] Barger JL (2008). A low dose of dietary resveratrol partially mimics caloric restriction and retards aging parameters in mice. PLoS ONE.

[4] Spindler SR, Mote PL, Flegal JM, Teter B (2013). Influence on longevity of blueberry, cinnamon, green and black tea, pomegranate, sesame, curcumin, morin, pycnogenol, quercetin, and taxifolin fed iso-calorically to long-lived, F1 hybrid mice. Rejuvenation Res.

[5] Miller BF, Robinson MM, Bruss MD, Hellerstein M, Hamilton KL (2012). A comprehensive assessment of mitochondrial protein synthesis and cellular proliferation with age and caloric restriction. Aging Cell.

[6] Um J-H (2010). AMP-activated protein kinase-deficient mice are resistant to the metabolic effects of resveratrol. Diabetes.

[7] Rodgers JT (2005). Nutrient control of glucose homeostasis through a complex of PGC-1alpha and SIRT1. Nature.

[8] Granner D, Pilkis S (1990). The genes of hepatic glucose metabolism. J. Biol. Chem..

[9] Salminen A, Kaarniranta K (2012). AMP-activated protein kinase (AMPK) controls the aging process via an integrated signaling network. Ageing Research Reviews.

[10] Zhou Y, Rui L (2010). Major urinary protein regulation of chemical communication and nutrient metabolism. Vitamins and hormones.

[11] Giller K, Huebbe P, Doering F, Pallauf K, Rimbach G (2013). Major urinary protein 5, a scent communication protein, is regulated by dietary restriction and subsequent re-feeding in mice. Proceedings. Biological sciences.

[12] Dhahbi JM, Kim HJ, Mote PL, Beaver RJ, Spindler SR (2004). Temporal linkage between the phenotypic and genomic responses to caloric restriction. Proc. Natl. Acad. Sci. USA.

[13] Baur JA (2006). Resveratrol improves health and survival of mice on a high-calorie diet. Nature.

[14] Svensson K (2015). Resveratrol and SRT1720 elicit differential effects in metabolic organs and modulate systemic parameters independently of skeletal muscle peroxisome proliferator-activated receptor gamma co-activator 1alpha (PGC-1alpha). J. Biol. Chem..

[15] Lagouge M (2006). Resveratrol improves mitochondrial function and protects against metabolic disease by activating SIRT1 and PGC-1alpha. Cell.

[16] Fusser M (2011). Spontaneous mutagenesis in Csb(m/m)Ogg1(−)(/)(−) mice is attenuated by dietary resveratrol. Carcinogenesis.

[17] Franco SS (2014). Resveratrol accelerates erythroid maturation by activation of FoxO3 and ameliorates anemia in beta-thalassemic mice. Haematologica.

[18] Flachsbart F (2017). Identification and characterization of two functional variants in the human longevity gene FOXO3. Nature communications.

[19] Essaghir A, Dif N, Marbehant CY, Coffer PJ, Demoulin JB (2009). The transcription of FOXO genes is stimulated by FOXO3 and repressed by growth factors. J. Biol. Chem..

[20] Tung BT (2015). Anti-inflammatory effect of resveratrol in old mice liver. Experimental gerontology.

[21] Guschlbauer M (2013). trans-Resveratrol and epsilon-viniferin decrease glucose absorption in porcine jejunum and ileum *in vitro*. *Comparative biochemistry and physiology*. Part A, Molecular & integrative physiology.

[22] Qiao Y (2014). Effects of resveratrol on gut microbiota and fat storage in a mouse model with high-fat-induced obesity. Food & function.

[23] Nikolai S, Pallauf K, Huebbe P, Rimbach G (2015). Energy restriction and potential energy restriction mimetics. Nutrition research reviews.

[24] Ingram DK (2006). Calorie restriction mimetics: an emerging research field. Aging Cell.

[25] Minor RK (2010). Chronic ingestion of 2-deoxy-D-glucose induces cardiac vacuolization and increases mortality in rats. Toxicol. Appl. Pharmacol..

[26] Heitman J, Movva NR, Hall MN (1991). Targets for cell cycle arrest by the immunosuppressant rapamycin in yeast. Science.

[27] EFSA-NDA-Panel (2016). Safety of synthetic trans-resveratrol. EFSA Journal.

[28] Miller RA (2011). Rapamycin, but not resveratrol or simvastatin, extends life span of genetically heterogeneous mice. J. Gerontol. A Biol. Sci. Med. Sci..

[29] Nakagawa S, Lagisz M, Hector KL, Spencer HG (2012). Comparative and meta-analytic insights into life extension via dietary restriction. Aging Cell.

[30] Pearson KJ (2008). Resveratrol delays age-related deterioration and mimics transcriptional aspects of dietary restriction without extending life span. Cell Metabolism.

[31] van der Made SM, Plat J, Mensink RP (2015). Resveratrol does not influence metabolic risk markers related to cardiovascular health in overweight and slightly obese subjects: a randomized, placebo-controlled crossover trial. PLoS ONE.

[32] Timmers S (2011). Calorie restriction-like effects of 30 days of resveratrol supplementation on energy metabolism and metabolic profile in obese humans. Cell Metabolism.

[33] Bode LM (2013). *In vivo* and *in vitro* metabolism of trans-resveratrol by human gut microbiota. Am. J. Clin. Nutr..

[34] National Diabetes Statistics Report. Centers for Disease Control and Prevention, U.S. Dept of Health and Human Services, Atlanta, GA (2017).

[35] Neuhauser H (2016). Hypertension in Germany. Dtsch Arztebl International.

[36] Tamayo T, Brinks R, Hoyer A, Kuß O, Rathmann W (2016). The Prevalence and Incidence of Diabetes in Germany: An Analysis of Statutory Health Insurance Data on 65 Million Individuals From the Years 2009 and 2010. Dtsch Arztebl International.

[37] Kopelman PG (2000). Obesity as a medical problem. Nature.

[38] Ozturk E, Arslan AKK, Yerer MB, Bishayee A (2017). Resveratrol and diabetes: A critical review of clinical studies. Biomedicine & pharmacotherapy = Biomedecine & pharmacotherapie.

[39] Schneider K, Oltmanns J, Hassauer M (2004). Allometric principles for interspecies extrapolation in toxicological risk assessment–empirical investigations. Regulatory toxicology and pharmacology.

[40] Faragher RG (2011). Resveratrol, but not dihydroresveratrol, induces premature senescence in primary human fibroblasts. Age (Dordr).

[41] Krauss J, Kopp U, Bracher F (2015). Short microwave-assisted modular synthesis of naturally occurring oxygenated bibenzyls. Zeitschrift für Naturforschung B.

[42] R-Core-Team, A language and environment for statistical computing (2015).

[43] Verbeke, G. A. M. Geert. *Linear mixed models for longitudinal data*. (Springer-Verlag, 2000).

[44] Laird NM, Ware JH (1982). Random-effects models for longitudinal data. Biometrics.

[45] Cochran WG (1957). Analysis of covariance: Its nature and uses. Biometrics.

[46] Weindruch R, Walford RL, Fligiel S, Guthrie D (1986). The retardation of aging in mice by dietary restriction: longevity, cancer, immunity and lifetime energy intake. J. Nutr..

[47] Bretz, F., Hothorn, T. & Westfall, P. *Multiple comparisons using R*. (CRC Press, 2010).

[48] Barger JL, Kayo T, Pugh TD, Prolla TA, Weindruch R (2008). Short-term consumption of a resveratrol-containing nutraceutical mixture mimics gene expression of long-term caloric restriction in mouse heart. Experimental Gerontology.

[49] Staats, S. *et al*. Dietary resveratrol does not affect life span, body composition, stress response, and longevity-related gene expression in Drosophila melanogaster. *International journal of molecular sciences* **19**, 10.3390/ijms19010223 (2018). 10.3390/ijms19010223 PMC5796172 29324667

[50] Thazhath SS (2016). Administration of resveratrol for 5 wk has no effect on glucagon-like peptide 1 secretion, gastric emptying, or glycemic control in type 2 diabetes: a randomized controlled trial. Am. J. Clin. Nutr..

[51] Cawthorn WP (2014). Bone marrow adipose tissue is an endocrine organ that contributes to increased circulating adiponectin during caloric restriction. Cell metabolism.

[52] Dourson ML, Stara JF (1983). Regulatory history and experimental support of uncertainty (safety) factors. Regulatory toxicology and pharmacology: RTP.

[53] Mukkavilli, R. *et al*. Absorption, metabolic stability, and pharmacokinetics of ginger phytochemicals. *Molecules* **22**, 10.3390/molecules22040553 (2017). 10.3390/molecules22040553 PMC6154694 28358331

[54] Marier J-F (2002). Metabolism and disposition of resveratrol in rats: extent of absorption, glucuronidation, and enterohepatic recirculation evidenced by a linked-rat model. J. Pharmacol. Exp. Ther..

[55] Rucker R, Storms D (2002). Interspecies comparisons of micronutrient requirements: metabolic vs. absolute body size. J. Nutr..

[56] Zamora-Ros, R. *et al*. Concentrations of resveratrol and derivatives in foods and estimation of dietary intake in a Spanish population: European Prospective Investigation into Cancer and Nutrition (EPIC)-Spain cohort. *British Journal of Nutrition* **100**, 10.1017/s0007114507882997 (2008). 10.1017/S0007114507882997 18096094

[57] Williams LD, Burdock GA, Edwards JA, Beck M, Bausch J (2009). Safety studies conducted on high-purity trans-resveratrol in experimental animals. Food Chem. Toxicol..

[58] Klinger, S. & Breves, G. Resveratrol inhibits porcine intestinal glucose and alanine transport: potential roles of Na(+)/K(+)-ATPase activity, protein kinase A, AMP-activated protein kinase and the association of selected nutrient transport proteins with detergent resistant membranes. *Nutrients* **10**, 10.3390/nu10030302 (2018). 10.3390/nu10030302 PMC5872720 29510506

[59] Yonamine CY (2016). Resveratrol improves glycemic control in insulin-treated diabetic rats: participation of the hepatic territory. Nutrition & metabolism.

[60] Lescale-Matys L (1993). Regulation of the ovine intestinal Na+/glucose co-transporter (SGLT1) is dissociated from mRNA abundance. Biochem. J..

[61] Vernaleken A (2007). Tripeptides of RS1 (RSC1A1) inhibit a monosaccharide-dependent exocytotic pathway of Na+ -D-glucose cotransporter SGLT1 with high affinity. J. Biol. Chem..

[62] Casirola DM, Rifkin B, Tsai W, Ferraris RP (1996). Adaptations of intestinal nutrient transport to chronic caloric restriction in mice. The American journal of physiology.

[63] Higashida K (2013). Effects of resveratrol and SIRT1 on PGC-1alpha activity and mitochondrial biogenesis: a reevaluation. PLoS Biol..

[64] Sharrow SD, Vaughn JL, Zidek L, Novotny MV, Stone MJ (2002). Pheromone binding by polymorphic mouse major urinary proteins. *Protein science: a publication of the Protein*. Society.

[65] Hurst JL (2001). Individual recognition in mice mediated by major urinary proteins. Nature.

[66] Zhou Y, Jiang L, Rui L (2009). Identification of MUP1 as a regulator for glucose and lipid metabolism in mice. J. Biol. Chem..

