# Supplementary material for: Resveratrol, lunularin and dihydroresveratrol do not act as caloric restriction mimetics when administered intraperitoneally in mice
Source: Sci Rep. 2019 Mar 14;9:4445. doi: 10.1038/s41598-019-41050-2 (PMC6418094; doi:10.1038/s41598-019-41050-2)
Supplement: Supplementary file 1 — Supplementary data, methods and figures [file 41598_2019_41050_MOESM1_ESM.pdf]

Supplemental material for the article:

Resveratrol, lunularin and dihydroresveratrol do not act as caloric restriction mimetics when administered intraperitoneally in mice

Kathrin Pallauf[1]*, Dawn Chin[1], Ilka Günther[1], Marc Birringer[2], Kai Lüersen[1], Gerald Schultheiß[3], Sarah Vieten[3], Jürgen Krauß[4], Franz Bracher[4], Nicolas Danylec[5], Sebastian T. Soukup[5], Sabine E. Kulling[5], Gerald Rimbach[1]

1- Institute of Human Nutrition and Food Science, University of Kiel, Hermann-Rodewald-Straße 6, 24118 Kiel, Germany

2- Department of Nutritional, Food and Consumer Sciences, Fulda University of Applied Sciences, Leipziger Straße 123, 36037 Fulda, Germany

3- Animal welfare office, University of Kiel, Olshausenstraße 40, 24118 Kiel, Germany

4- Department of Pharmacy – Center for Drug Research, Ludwig-Maximilians University, Butenandtstraße 5-13, 81377 Munich, Germany

5- Max Rubner-Institut, Federal Research Institute of Nutrition and Food, Department of Safety and Quality of Fruit and Vegetables, Haid-und-Neu-Straße 9, 76131 Karlsruhe, Germany

* corresponding author (email address: pallauf@foodsci.uni-kiel.de)

Supplemental Data

Supplemental Data X-1

Mouse weight and average feed intake during the injections with resveratrol, dihydroresveratrol, lunularin or DMSO/saline. Apart from the CR mice, the animals had free access to feed.

Group	Mean daily feed intake during i.p. injections [g]	Weight at beginning of trial [g]	Weight at beginning of i.p. injections [g]	Weight at end of trial [g]
AL-control (n=8)	3.00 ± 0.05	37.8 ±4.8	39.4 ±5.4	40.8 ± 6.1
CR-control[1] (n=8)	2.00 ± 0.06	38.1 ± 4.0	31.2 ± 3.8	25.7 ± 1.5
Resveratrol (n=9)	3.01 ± 0.10	37.9 ± 3.3	40.5 ± 3.8	41.2 ± 4.1
Dihydroresveratrol (n=10)	2.98 ± 0.10	37.8 ± 3.2	40.0 ± 3.9	41.3 ± 3.7
Lunularin (n=10)	2.81. ± 0.10*	37.8 ± 3.3	40.0 ± 3.2	38.8 ± 2.9.

[1]CR mice were fed 60% of what the AL-control consumed, however if the body weight of a mouse decreased more than 20 % compared to the initial bodyweight, up to 70% were fed until the mouse stopped losing weight. * $p < 0.05$ compared to the AL-control

Supplemental Data X-2.

Compound-dependent parameters and sMRM settings (AB Sciex QTrap 5500) for UHPLC-MS/MS analyses of resveratrol and metabolites in mouse liver samples

	Transition	Mass 1	Mass 2	RT [min]	DP [V]	CE [V]	CXP [V]
Resveratrol	1	227.0	185.0	10.43	-98	-26	-10
	2	227.0	143.0	10.43	-98	-34	-6
Dihydro-resveratrol	1	229.0	123.0	10.65	-83	-22	-7
	2	229.0	81.0	10.65	-83	-36	-10
Lunularin	1	213.1	107.0	13.86	-90	-22	-6
	2	213.1	106.0	13.86	-90	-22	-6
$^{13}C_6$-Resveratrol	1	233.0	191.0	10.43	-94	-26	-11
	2	233.0	149.0	10.43	-94	-36	-8

MRM detection window: 60 s; target scan time: 0.3 s; transition 1: quantifier; transition 2: qualifier; RT: retention time; DP: declustering potential; CE: collision energy voltage; CXP: collision cell exit potential.

Supplemental Data X-3

Validation results for UHPLC-MS/MS analyses of resveratrol and metabolites in mouse liver samples

Selectivity

Resveratrol, dihydroresveratrol and lunularin were identified in each case by retention time and two MS/MS transitions. Mouse liver samples from resveratrol-free mice showed no interfering peaks in chromatograms at the specific retention times.

Accuracy, intra-day precision and recovery

	**Accuracy [%]**	**Intra-day precision [%]**	**Recovery ± SD [%]**
Resveratrol	102	6.0	70.7 ± 5.4
Dihydroresveratrol	104	4.3	71.4 ± 4.5
Lunularin	102	13.3	57.9 ± 4.8

(500 pmol/g liver, n = 6; SD, standard deviation)

Linearity

A best fit line was obtained by linear regression using a weighting of $1/x^2$. The correlation coefficient was used as an indicator for the quality of the calibration curves and was >0.9944.

Limit of quantitation (LOQ) and limit of detection (LOD)

LOQ and LOD were defined as the levels with a signal-to-noise (S/N) ratio of 10 and of 3, respectively.

	**Limit of quantitation (LOQ) [pmol/g liver]**	**Limit of detection (LOD) [pmol/g liver]**
Resveratrol	6.8	2.0
Dihydroresveratrol	7.5	2.3
Lunularin	32.3	9.7

Supplemental Methods

Analysis of resveratrol and metabolites in mouse liver samples

The UHPLC-MS/MS analysis and the sample preparation for these analyses took place at the MRI, Karlsruhe, Germany. Resveratrol (>99%) for UHPLC-MS/MS analyses was purchased from Sigma Aldrich while dihydroresveratrol (>99.5%) was purchased from LKT Laboratories (St. Paul, Minnesota, USA). Lunularin was synthesized as described in Experimental Procedures. $^{13}C_6$- resveratrol (chemical purity: 98.9%, isotopic purity: 98.7%) was purchased from Toronto Research Chemicals (North York, Canada). All other chemicals and solvents used were of analytical grade. Deionized water was taken from an in-house ultrapure water system (LaboStar; Siemens, Erlangen, Germany) with a conductivity of 0.055 µS/cm.

For sample preparation mouse livers were homogenized with a ball mill (MM400; Retsch, Haan, Germany). Before starting the homogenization, liver samples and the grinding beakers including grinding balls were pre-cooled with liquid nitrogen. Homogenized liver samples were portioned in approx. 100 mg aliquots and stored at -80°C until analysis. The sample preparation procedure was done on ice wherever possible. Quantification of the analytes was done by external calibration. Therefore analyte-free blank mouse liver samples were spiked with 5 µL of internal standard (10 µM of $^{13}C_6$- resveratrol in DMSO) and 5 µL of analyte standard solutions in DMSO (resveratrol, dihydroresveratrol and lunularin each in an end level range between 4 and 2500 pmol/g liver).

Aliquots of mouse liver samples were spiked with 5 µL of internal standard (10 µM of $^{13}C_6$- resveratrol in DMSO) and 5 µL of DMSO. Afterwards 700 µL of buffer A (0.1 M ammonium acetate buffer, pH 5, 10 mM DL-dithiothreitol) were pipetted to the samples, followed by 50 µL β-glucuronidase (25 U/µL, bovine liver B-3; Sigma Aldrich) and 50 µL sulfatase (1.875 U/µL, *Helix pomatia* H-1; Sigma Aldrich, Taufkirchen, Germany) both dissolved in buffer A. Then samples were vortexed for 10 s and incubated for 2 h at 37°C and 650 U/min (Thermomixer Comfort; Eppendorf, Wesseling-Berzdorf, Germany). Afterwards, samples were immediately put on ice and centrifuged at 23,100 x g and 4°C for 5 min. The supernatants were transferred to new sample tubes. The residues were extracted twice by adding 800 µL of 50% (v/v) methanol and 1% (v/v) formic acid in water, followed by vortexing (10 s), centrifugation (23,100 x g, 4°C, 5 min) and transferring of supernatants to the extracts. Combined extracts were diluted with 5 mL of water and cleaned up by solid phase extraction (SPE). Therefore SPE tubes (30 mg/1 mL Strata-X; Phenomenex, Aschaffenburg, Germany) were first conditioned with 1 mL of methanol and then equilibrated with 1 mL of 0.1% (v/v) formic acid in water. After loading with the sample solutions, the tubes were washed twice with 1 mL of 0.1% (v/v) formic acid in water, followed by 2-times 1 mL of 5% (v/v) methanol in water. The analytes were eluted twice with 1 mL of methanol and evaporated to dryness under a nitrogen stream. The residues were dissolved in 100 µL of 30%

methanol (v/v) in water and centrifuged at 16,000 x g and 4°C for 5 min. The supernatants (80 µL) were transferred to HPLC vials and analyzed by UHPLC-MS/MS.

The UHPLC-MS/MS analyses were performed on an QTrap 5500 mass spectrometer (AB Sciex, Darmstadt, Germany) equipped with a Nexera LC system (Shimadzu, Duisburg, Germany), which consisted of a controller (CBM-20A), a degasser (DGU-20A5), two pumps (LC-30AD), an autosampler (SIL-30AC), a column oven (CTO-20AC) and a DAD (SPD-M20A). The system was controlled by the software Analyst 1.6.2. Separation of the analytes was achieved on a Waters Acquity HSS T3 (2.1 mm x 100 mm, 1.8 µm particle size) equipped with a pre-column (Waters Acquity HSS T3, 2.1 mm x 5 mm, 1.8 µm particle size) and a pre-in-line filter (Phenomenex Krudkatcher, 0.5 µm). The column oven temperature was adjusted to 40 °C. Solvent A was an aqueous 25 mM ammonium formate buffer with 0.1% (v/v) formic acid and solvent B was acetonitrile. The flow rate was set to 0.5 mL/min. The injected sample volume was 10 µL. The gradient elution profile was as follows: 0.0-2.0 min isocratic with 3% B, 2.0-15.0 min from 3 to 45% B, 15.0-17.0 min from 45 to 95% B, 17.0-18.5 min isocratic with 95% B, 18.5-20.0 min from 95 to 3% B, and 20.0-23.0 min isocratic at the initial conditions. To minimize contamination of the ion source, the eluate was only transferred to the MS at the time when the analytes of interest eluted from the column.

The turbo spray ESI source of the MS was operated in the negative mode. The source parameters were as follows: curtain gas (CUR) 45 psi, ion spray voltage (IS) -4,500 V, ion source gas-1 (GS 1) 70 psi, ion source gas-2 (GS 2) 70 psi, ion source gas-2 temperature (TEM) 600 °C. The quadrupole detectors Q1 and Q3 were operated at unit resolution in the scheduled multiple-reaction monitoring (sMRM) mode using two transitions (a quantifier and a qualifier ion transition for each compound) for unambiguous identification of each analyte. The sMRM experiments with the associated settings are summarized in Supplemental Data X-2. Nitrogen was used as collision gas.

Analysis of UHPLC-MS/MS data was performed with MultiQuant 2.1.1 software (ABSciex, Darmstadt, Germany). To obtain the calibration curves, the ratios of the analyte peak areas to the internal standard peak area were calculated and plotted against the analyte level. A best fit line was obtained by linear regression using a weighting of $1/x^2$.

The method was validated based on FDA criteria. In detail, selectivity, accuracy, intra-day precision, recovery, linearity, limit of quantitation (LOQ) and limit of detection (LOD) were determined. The validation results are summarized in Supplemental Data X-3.

Statistics

The statistical software R [36] was used to evaluate the data. Appropiate models were defined [37,38]. This was a linear model for the blood parameters, feed intake, blood parameters and Ussing chamber

measurements and a mixed model for the PCRs. Here, the treatment group was regarded as a fixed factor and the PCR run was regarded as a random factor. For the weekly weight development, the statistical model included the treatment group (CON, RSV, DHR, LUN), the covariate 'week' as well as their interaction term as fixed factors. The mouse was regarded as random factor. We excluded the CR mice from the statistical analysis of feed uptake and bodyweight.

The data was assumed to be approximately normally distributed. For the weight development, the data was assumed to be heteroscedastic due to the week and treatment group. These assumptions are based on a graphical residual analysis. Based on this model, a pseudo R^2 was calculated [62]. While for the blood parameters, feed intake and normalized mRNA levels an analysis of variances (ANOVA) was conducted, an analysis of covariances (ANCOVA) was conducted for the weight development[39,40]. These analyses were followed by multiple contrast tests (Dunnett) [41] to compare the blood levels, feed intake, ISC values, normalized mRNA expressions and slopes.

Supplemental Figures

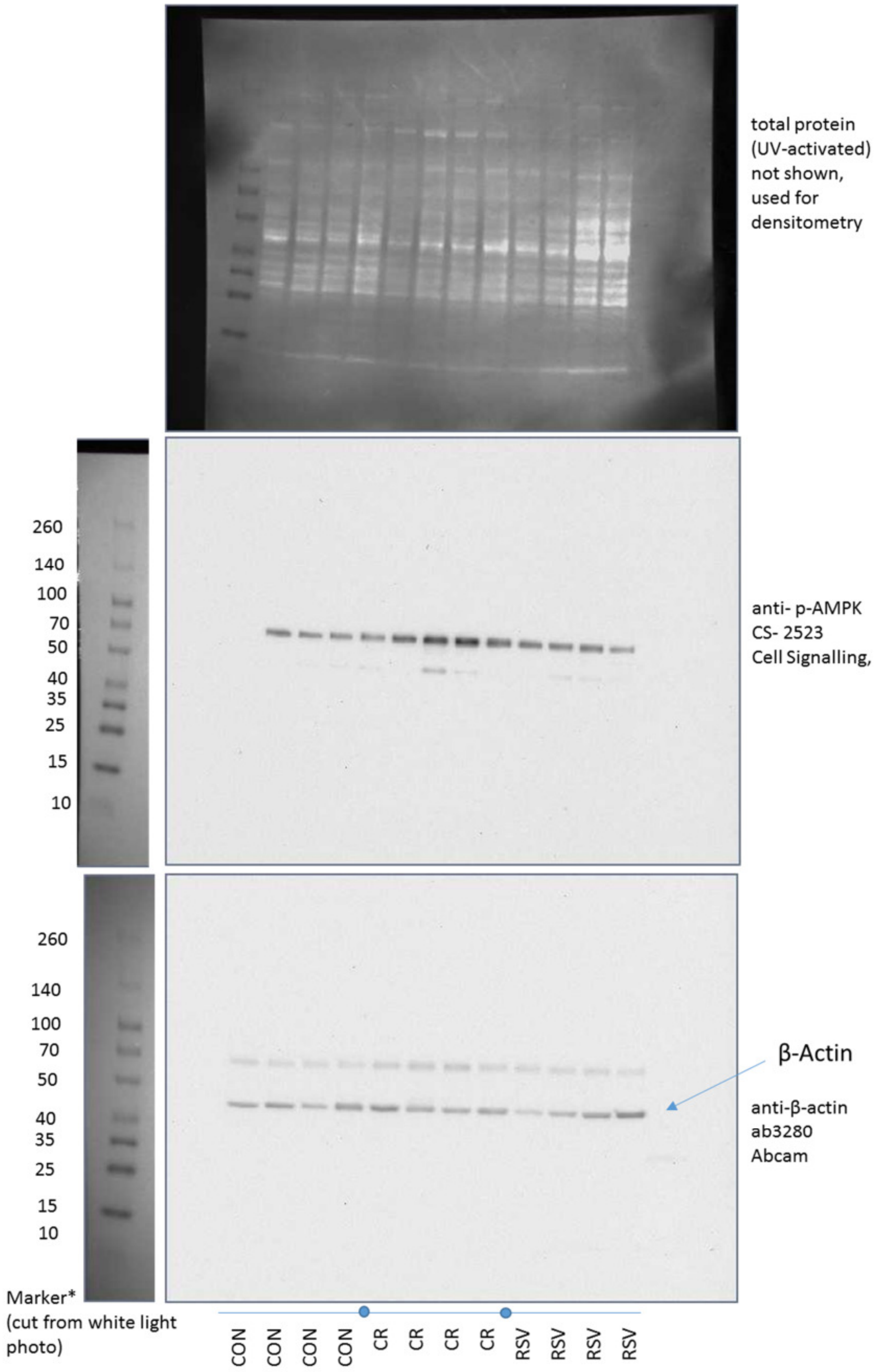

* Spectra TM Multicolor Broad Range Protein Ladder Thermo Scientific 26634, Waltham, USA

Full-length images Figure 5a left

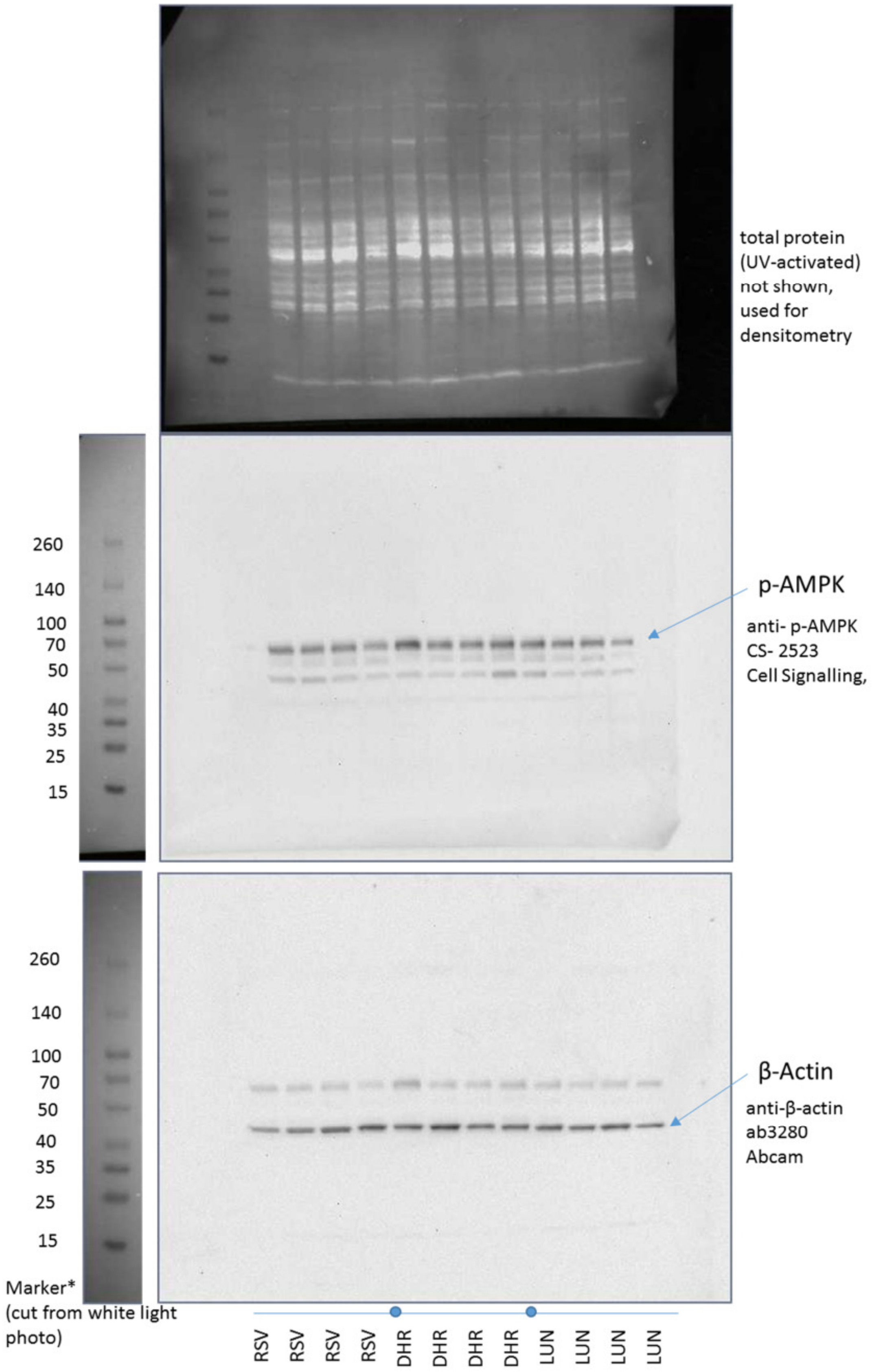

* Spectra TM Multicolor Broad Range Protein Ladder Thermo Scientific 26634, Waltham, USA

Full-length images Figure 5a right

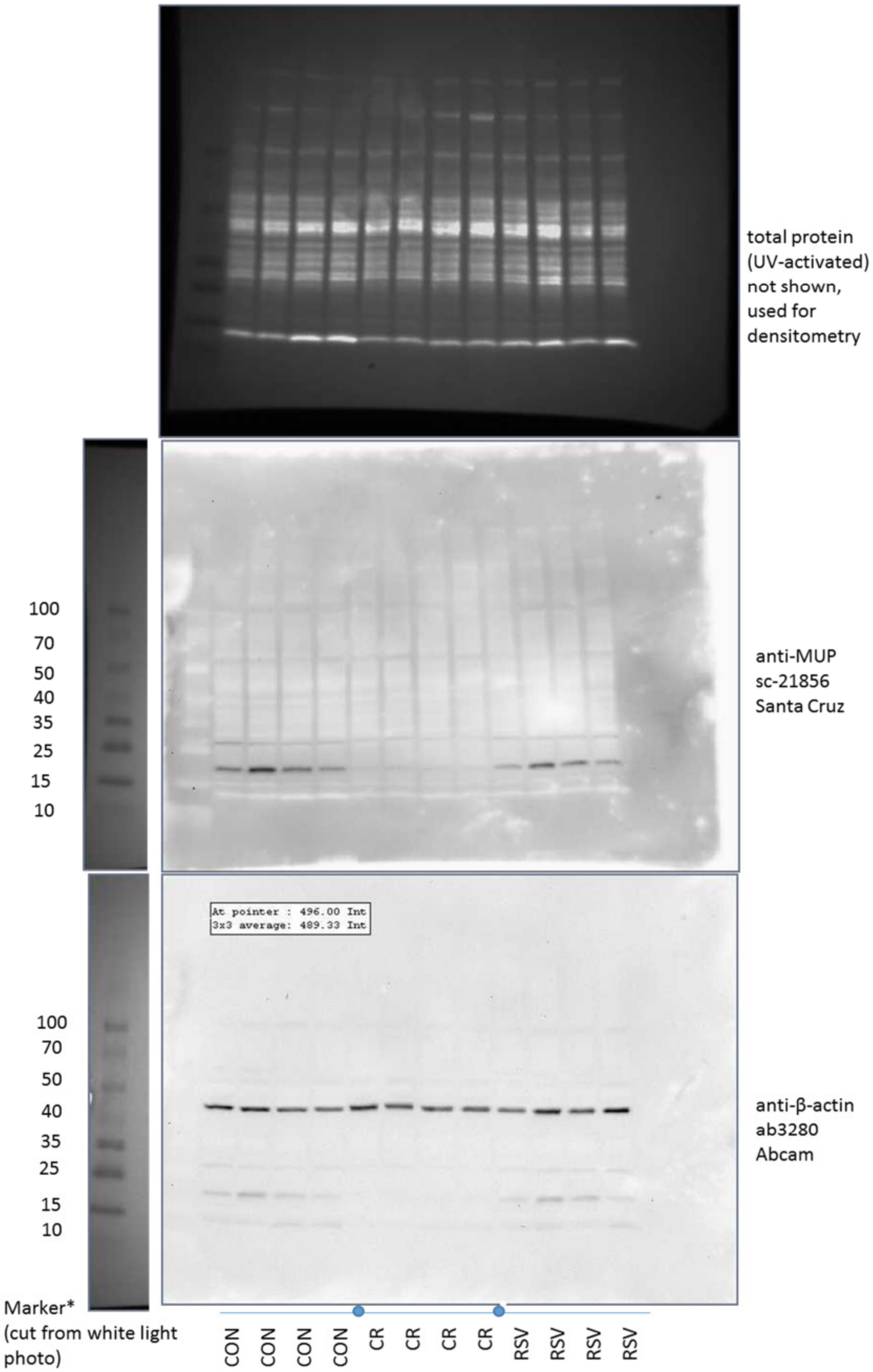

* Spectra TM Multicolor Broad Range Protein Ladder Thermo Scientific 26634, Waltham, USA

Full-length images Figure 5b left

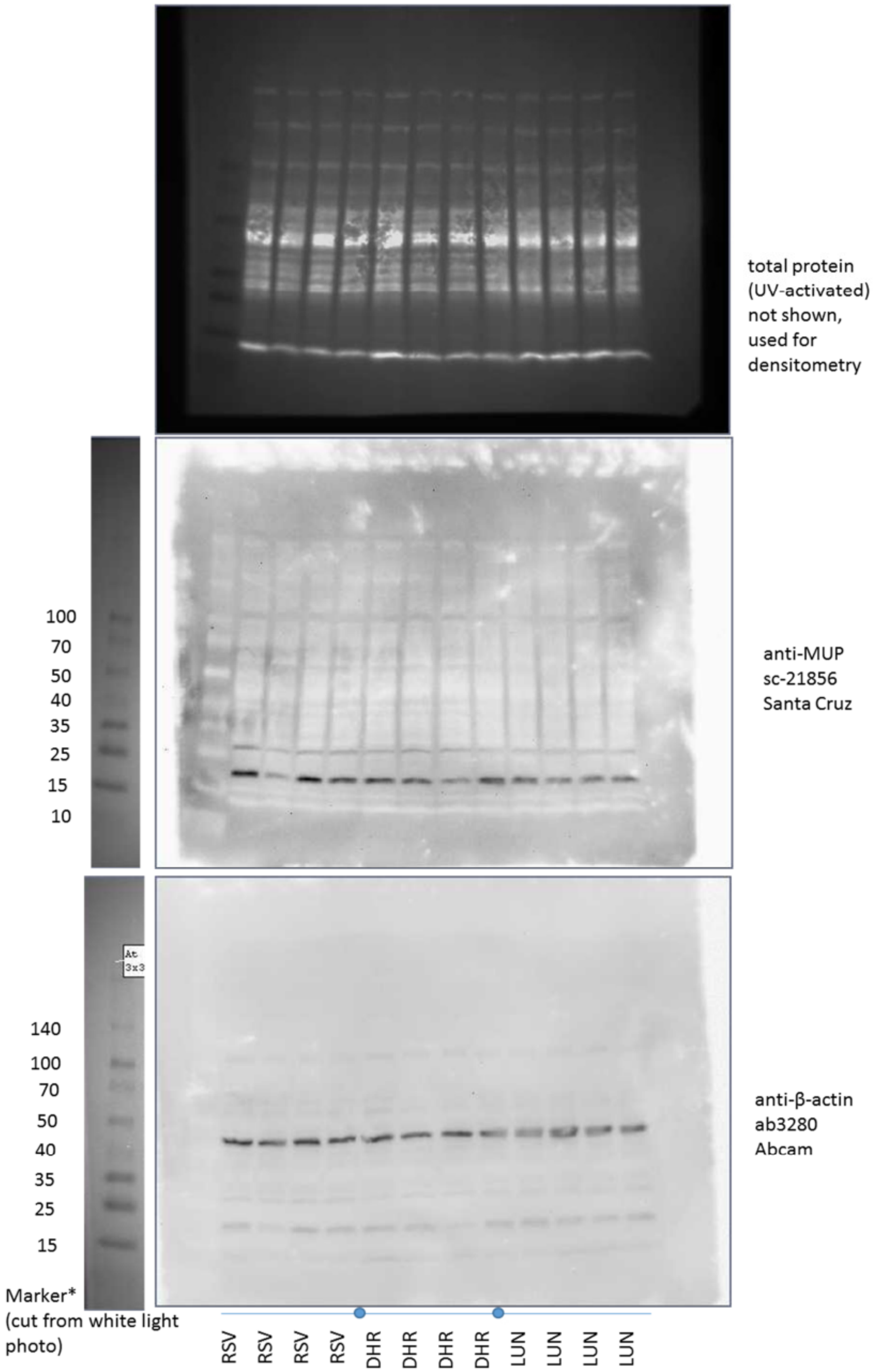

* Spectra TM Multicolor Broad Range Protein Ladder Thermo Scientific 26634, Waltham, USA

Full-length images Figure 5b right
